# Chains of tragedy: The impact of bullying victimization on mental health through mediating role of aggressive behavior and perceived social support

**DOI:** 10.3389/fpsyg.2022.988003

**Published:** 2022-11-08

**Authors:** Yi Guo, Xiao Tan, Qiu-jin Zhu

**Affiliations:** ^1^Institute of Educational Sciences, Hubei University of Education, Wuhan, China; ^2^School of Philosophy, Wuhan University, Wuhan, China

**Keywords:** bullying victimization, mental health, aggressive behavior, perceived social support, chain mediation effect

## Abstract

**Objective:**

Bullying is a worldwide concern for its devastating consequences. The current study focused on bullying victims, examining the effects of being bullied on mental health and the chain of mediating mechanisms among adolescents. Specifically, this study attempts to explain the relationship between bullying victimization and mental health from the perspective of maladaptive behavior and perceived social support.

**Methods:**

A total of 3,635 adolescents responded to questions on bullying victimization, aggressive behavior, perceived social support, and mental health measurements including anxiety, depression, and subjective well being scale combined.

**Results:**

(1) Bullying victimization was significantly correlated with aggressive behavior, perceived social support, and mental health, including anxiety, depression, and subjective well being. (2) Bullying victimization not only negatively predicts mental health levels but also has an indirect impact on mental health through three pathways: a separate mediating effect on aggressive behavior, a separate mediating effect on perceived social support, and a chain mediating effect on both.

**Conclusion:**

The present results demonstrate that maladaptive behavior by bullying victims can lead to changes in their perceived social support and mental health problems. Violence begets violence and provides no constructive solutions, instead, produces a tragic chain of victimization. Further implications are discussed accordingly.

## Introduction

Bullying victimization is defined as being the target of unwanted aggression and harm in various forms, such as verbal, physical, relational, social bullying, and electronic bullying (Olweus, 1993; Sun and Shi, 2017). Bullying is a universal phenomenon across different cultures (Chan and Wong, 2015; Liu and Lu, 2017). Globally, 246 million children reported experiencing bullying and school violence annually (UNESCO, 2019). Numerous studies have verified that being bullied has devastating consequences (Peng et al., 2020). Those victims of bullying are at an increased risk of low self-confidence, emotional impairment, low level of well being, poor mental health, and even attempts of suicide in both Western and Eastern countries (Cosma et al., 2017; Shaheen et al., 2019).

Bullying victimization generally leads to a lower level of mental health quality, and this relationship is also influenced by the victims' coping strategy, including the cognition and behavior an individual employs to reduce distress/tension or eliminate stressors (Scarpa and Haden, 2006). Previous research has explored bullying victims' coping strategies and the consequences, such as the use of humor, cognitive coping strategies, and help-seeking (Newman et al., 2011; Garnefski and Kraaij, 2014; Nixon et al., 2020; Xie et al., 2022). However, previous studies do not adequately consider the multiple coping strategies of bullying victims simultaneously nor examine the underlying relationship among these mechanisms.

According to the general aggression model (GAM), the experience of being bullied as a passive situational factor influences the likelihood of aggressive behavior by exerting influence on aggressive thoughts, angry feelings, and arousal levels, as well as the related appraisal and decision processes. Aggressive action as an outcome influences the social encounter, which usually causes negative social consequences, such as others' responding to the aggression, acting in retaliation, or staying away from the aggressor. At this point, when the individual's reappraisal process is activated, it can influence the present internal state variables (Anderson and Bushman, 2002; Allen et al., 2018). And the support deterioration model states that stressful events like being bullied deteriorate the perceived availability or the effectiveness of social support, which leads to mental health problems (Barrera, 1986). Thus, the current research investigates multiple coping strategies that affect the mental health of bullying victims, and the underlying relationship among these mechanisms. Specifically, this study attempts to examine bullying victims who conduct maladaptive behavior that would lead to a change in their perceived social support and then the level of mental health.

### The experience of being bullied harms mental health

Bullying victims are at elevated risk for various externalizing and internalizing problems (Loukas and Pasch, 2013). The research found that different forms of bullying (physical, relational, verbal, and cyber) are associated with different harmful behaviors (self-harm, suicide attempts, and suicidal ideation) (Sinclair et al., 2022). Being bullied may also result in serious psychological maladjustment and emotional maladaptation. Being the target of bullying leads to the development of hostile attributions and internalizing negative peer messages, and victimization triggers internalized issues in individuals, such as anxiety, depression, low self-esteem, and feelings of loneliness (Loukas and Pasch, 2013; Cross et al., 2015).

In recent years, researchers started to examine the impact of bullying on individual well being (Varela et al., 2018; Miranda et al., 2019). A cross-country study of 47,029 children and adolescents in 15 countries found that bullying had a significant negative impact on subjective well being across countries and at different ages (Savahl et al., 2019). In China, 636 boarding students of grades 4–6 in rural primary schools were investigated by questionnaire, including school bullying, subjective well being, school bonding, and positive psychological capital, and school bullying was negatively correlated with school bonding and subjective well being (Wu et al., 2022).

The dual-factor model of mental health suggests that the concept of a good mental health condition includes the absence of negative indicators (e.g., depression, anxiety, negative affect) and the presence of positive indicators (e.g., subjective well being, life satisfaction, positive affect), which is a more comprehensive and accurate assessment of individual mental health (Greenspoon and Saklofske, 2001; Suldo and Shaffer, 2008). And based on the above analysis, this study proposes **H1**: Bullying victimization negatively predicts mental health levels, including levels of anxiety, depression, and subjective well being.

### The impact of bullying victimization on mental health through the mediation effect of maladaptive behavior

Several studies have found victimization increases the risk of maladaptive behavior. Individuals who have suffered from bullying usually have no reasonable way to resolve the accumulation of psychological problems, such as panic, social anxiety, and depression, and this can generate explosive attacks and illegal anti-social behavior (Li, 2016; Liu and Lu, 2017). The longitudinal research from different cultures also showed that victimization has a long-term negative impact and produces maladaptive reactions. Bullying victims exhibit obstacles in interpersonal communication and produce behavior deviation (Liu and Zhao, 2013), and the experience of being bullied could significantly predict aggressive behavior, taking revenge, and getting involved in illegal and violent crimes and violent crimes in adulthood (Jackson et al., 2013; DeCamp and Newby, 2015).

Maladaptive behavior of bullying victims could produce mental health issues. Bullying victims scored higher on hostile interpretation, anger, retaliation, and ease of aggression than the other children (Camodeca and Goossens, 2005), and aggressive acts that would occur as impulsive behavior to cause harm to the source of frustration and defend themselves were positively associated with generalized anxiety symptoms and depressive symptoms (Pederson et al., 2018).

The experience of being bullied, maladaptive behavior, and mental health appeared to be closely linked. The bullying victims who engage in aggressive behavior are more likely to attribute hostile intent in ambiguous situations and react more aggressively to peer conflict, which further elicits peer rejection and behavior problems, and are the most maladapted and in greatest need of intervention (Bettencourt et al., 2013). Studies have found evidence that depression, anxiety, and loneliness were characteristics of aggressive victims (Shao et al., 2014); however, there is also some data which provide no evidence of unique social-emotional dysfunction of aggressive victims. Thus, properly accounting for potentially confounding influences on the internalizing problems is needed (O'Connor et al., 2019; O'Connor, 2021). The impact of victimization on mental health through maladaptive behavior needs to be examined further. As a result, this study proposes Hypothesis **H2**: Bullying victimization can impact mental health levels through the mediating role of aggressive behavior.

### The impact of bullying victimization on mental health through perceived social support

Social support is defined as social interactions or relationships that provide individuals with the assistance or support that embed individuals within a social system to provide love, care, or a sense of attachment to a valued social group. And perceived social support is the belief that these helping behaviors will occur when needed (Norris and Kaniasty, 1996). If individuals believe they are loved and valued and can depend on others, they are more likely to have help-seeking behavior (Lakey and Cohen, 2000).

Perceived social support may be viewed as a variable that has wide-ranging effects on physical and mental well being (Scarpa and Haden, 2006). Research reported that adolescents' perceived social support was significantly negatively correlated with suicidal ideation in cyberbullying victimization (Xu et al., 2021). The research which explored the perceived social support in crime victims proposed that chronic victimization erodes the victim's perception of social support and in turn, leads to heightened levels of distress and is detrimental to the victim's well being (Yap and Devilly, 2004).

Even worse, it has been found that most bullying victims do not actively report these experiences to parents and teachers, and do not seek help in the case of low perceived social support, which creates a vicious cycle of bullying and victimization (Haataja et al., 2016; Yablon, 2017). The data indicated that only one in four chronically victimized students turned to school staff for help, and 30% of bullying victims kept silent about their problems (Smith and Shu, 2000; Sitnik-Warchulska et al., 2021).

Thus, understanding the social context in which bullying occurs and the individual's perceived social support in different sources is vital to comprehend both the unique associations between bullying victims and mental health, and facilitate the development of prevention and intervention activities (Noret et al., 2020). Based on the above literature, this study proposes Hypothesis **H3**: Bullying victimization can impact mental health levels through the mediating role of perceived social support.

### The relationship between maladaptive behavior and perceived social support

Evidence suggests that the motivation for aggressive behavior is resorting to control or getting higher social status among peers, but it is the distorted relationship chains (Juvonen and Graham, 2014; Sun and Shi, 2017). The victim's aggressive behavior could be from mimicking the parents and other adults in the family or social setting (Bandura, 1974). Some of them are isolated from social groups during early childhood, forming interpersonal bonds through inappropriate behaviors, such as aggression (Olweus, 1978). Published data have identified that bullying victims with aggressive behavior do not share any social benefits with the high social status of bullies, but they have a higher level of distress and peer rejection (Juvonen et al., 2003; Chen and Zhang, 2018).

Meta-analysis summarizes that bullying victims who engage in aggressive behavior are concurrently associated with a range of adjustment difficulties, including loneliness, school-related fear, anxiety or avoidance, low self-esteem, and fear or avoidance of social interactions (Reijntjes et al., 2010). The feeling of neglect in peer relationships may impede their ability to build solid prosocial ties with others and limit their opportunities to gain sufficient social support. For instance, children engaging in bullying perpetration often reported a low quality of parental relationships, which are associated with further psychosocial difficulties in adolescent development (Sitnik-Warchulska et al., 2021). Another study in China also found a significantly negative correlation between bullying and perceived social support (Yang, 2020). The gradual alienation from peers may also impact bullying victims' mental health, such as anxiety and depression. On this basis, this study proposes Hypothesis **H4**: Aggressive behavior and perceived social support play a chain mediating role between bullying victimization and mental health.

**Figure 1:**
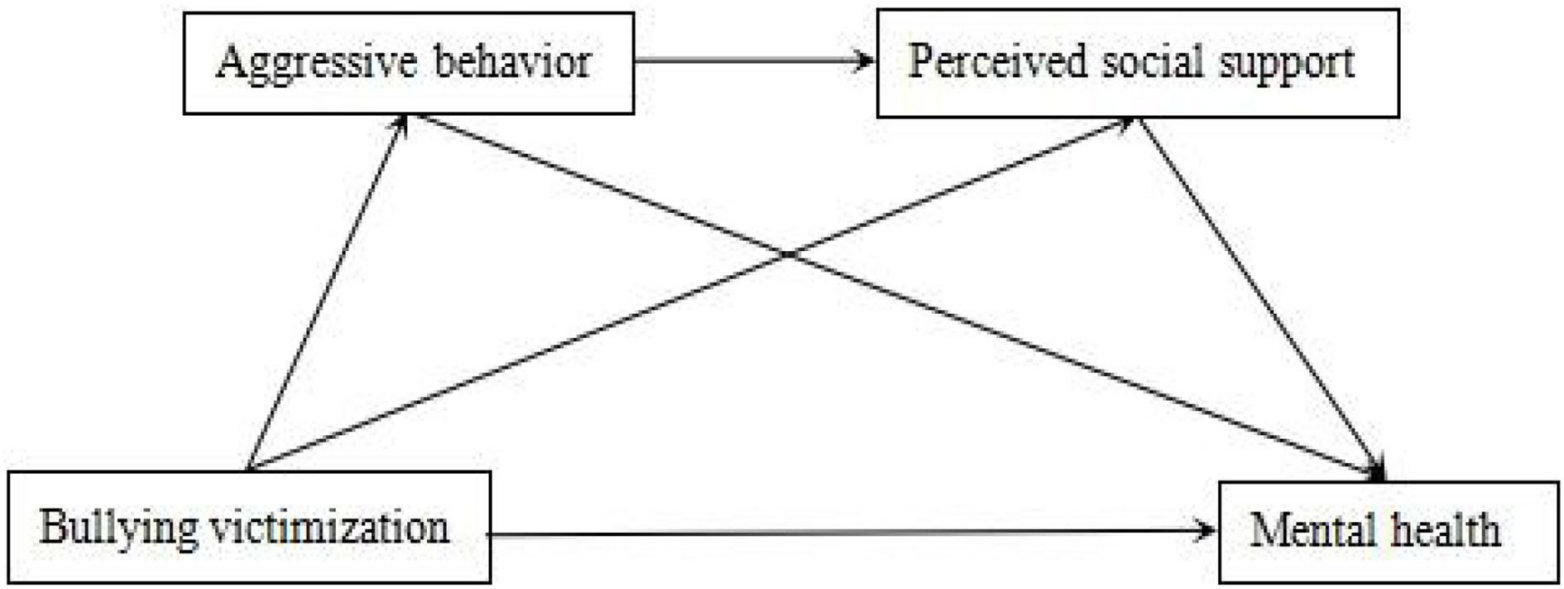
Relationship path map of bullying victimization, aggressive behavior, perceived social support, and mental health.

Therefore, the current study explores the impact of the experience of being bullied and their maladaptive behavior on mental health and whether perceived social support plays a vital role in the relationship. We investigated the chain mediating effect of bullying victims' aggressive behavior and perceived social support in the relationship between the experience of being bullied and mental health among adolescents. From the adaptation perspective to understand the perplexing role of bullying victims, we try to underline the mechanism of bullying victims' dysfunctional behavior and provide empirical evidence for intervention programs. The relationship path diagram proposed in this study is illustrated in Figure 1 as follows.

## Materials and methods

### Participants and procedure

Eight middle schools were randomly selected in the Hubei province of China. The research ethics committee approved the study at the Hubei University of Education, and it was conducted with the consent of the school and the adolescents' guardians. The students were told that none of their responses would be revealed to anyone and that they could stop participating at any time without penalty. All participants completed an online questionnaire in Chinese. A total of 3,635 valid online questionnaires were obtained. Among students who participated in the survey, 1,757 were male, and 1,878 were female, 968 were studying in grade junior one, 930 were in grade junior two, 583 were in grade junior three, and 547 were in grade senior one, and 607 were in grade senior two. The demographic information of the participants is shown in Table 1.

**Table 1:** Demographic information in the bullying victimization score of adolescents.

**Variables**	**All sample**	**Bullying victimization scores**	***t/F***	***p***
	**(*n* = 3,635)**	**(*M ±* SD)**	
**Gender**			*t =* −1.05	0.30
Male	1,757 (48.34%)	1.24 ±0.57		
Female	1,878 (51.66%)	1.26 ±0.52		
**Grade**				
Junior one (A)	968 (26.63%)	1.34 ±0.65	*F* = 8.24	<0.01
Junior two (B)	930 (25.58%)	1.22 ±0.50	A > B; A > C; A > E	
Junior three (C)	583 (16.04%)	1.18 ±0.42		
Senior one (D)	547 (15.05%)	1.26 ±0.53		
Senior two (E)	607 (16.70%)	1.22 ±0.52		
**Family structure**			*F* = 15.50	<0.01
Single parent (A)	598 (16.45%)	1.33 ±0.66	A > B; C > B	
Nuclear family (B)	2,548 (70.10%)	1.22 ±0.48		
Others (C)	489 (13.45%)	1.33 ±0.66		
**Parent education**			*F* (Father) = 12.35	<0.01
Father College or above (A)	328 (9.02%)	1.19 ± 0.48	C > A; C > B	
Father Senior high school (B)	1,432 (39.40%)	1.21 ± 0.47		
Father Junior high school or below (C)	1,875 (51.58%)	1.30 ± 0.60		
Mother college or above (A)	281 (7.73%)	1.21 ± 0.51	*F* (Mother) = 11.59	<0.01
Mother Senior high school (B)	1,271 (34.97%)	1.20 ± 0.45	C > A; C > B	
Mother Junior high school or below (C)	2,083 (57.30%)	1.29 ± 0.59		

### Measurements

#### Bullying victimization

The bullying victimization is measured by the Olweus Bully/Victimization Questionnaire (OBVQ). The Chinese version of OBVQ which was adopted in current research, was revised with good validity and reliability among adolescents (Xie et al., 2015). The OBVQ is comprised of three dimensions namely physical bullying, verbal bullying, and relational bullying. The scale has 12 items. Each item is rated on a six-point Likert scale ranging from 1 (never happened this semester) to 6 (happened every day this semester). The higher score of OBVQ represents a higher degree of victimization experience. In this study, the Cronbach's α value of the questionnaire was 0.93.

#### Aggressive behavior

The aggressive behavior was measured by a tool to measure aggressive behavior extracted from externalizing problem behavior for adolescents developed by Zhang et al. (2011). In this study, seven questions were selected and adapted as required, such as “fighting ”, “destroying public property or other people's property for no reason”, “verbally abusing others”, etc., using a five-point Likert scale. The subjects were asked to rate the frequency of the occurrence of these behaviors in the last six months. The mean scores of the seven items were calculated with higher scores indicating more aggressive behavior. Previous studies have shown that the questionnaire has good reliability and validity in the Chinese cultural context (Yu et al., 2011). In this study, the Cronbach's α value of the questionnaire was 0.71.

#### Perceived social support

Zimet et al. (1988) developed the Multidimensional Scale of Perceived Social Support (MSPSS) to measure the perceived adequacy of social support received from family, friends, and significant others. The MSPSS includes 12 items (e.g., “I can count on my friends when things go wrong”). Respondents report their agreement on a 7-point Likert-type scale with higher total score meaning higher perceived social support. Previous studies showed that the scale had a good reliability and validity when used with Chinese adolescents (Yang and Han, 2021). The Cronbach's α value of the Chinese MSPSS used in this study was 0.96.

#### Depression

The Chinese version of the 9-item Patient Health Questionnaire (PHQ-9) was used to measure the severity of depressive symptoms. A total score ranged from 0 to 27 (higher points indicating more severe depressive symptoms), with each item that can earn 0 to 3 points (0 = Not at all to 3 = Nearly every day). Depressive symptoms were classified by severity into five groups, namely, minimal (scores of 0-4), mild (5-9), moderate (10-14), moderately severe (15-19), and severe (20-27) (Kroenke et al., 2001). In this study, the average depression score was 3.05, and the standard deviation was 4.56, indicating that the participants' overall depressive symptoms were relatively mild. The Chinese version of PHQ-9 has been widely used, and previous studies showed that the scale had good reliability and validity when used with Chinese adolescents (Leung et al., 2020). In this study, the Cronbach's α value of the questionnaire was 0.93.

#### Anxiety

Anxiety symptoms were measured using the Chinese version of the Generalized Anxiety Disorder scale (GAD-7; Tong et al., 2016). Each item has four response options ranging from 0 to 3 (0 = Not at all to 3 = Nearly every day). Each participant can obtain a total score from 0 to 21, with higher score indicating more severe anxiety symptoms. Cut points of 5, 10, and 15 might be interpreted as representing mild, moderate, and severe levels of anxiety on the GAD-7 (Spitzer et al., 2006). In this study, the average depression score was 2.98, and the standard deviation was 3.94, indicating that the participants' overall depressive symptoms were relatively mild. The Chinese version of GAD-7 can be used in the Chinese context with good reliability and validity (Zeng et al., 2013; Tong et al., 2016). This scale had good internal consistency, with a Cronbach's α value of 0.93.

#### Subjective well being

Subjective well being was measured using the two components: Index of well being and Index of General Affect (Campbell, 1976). Ratings for each item in the overall index ranged from 1 to 7. The Index of General Affect consists of eight items that describe the connotation of emotions at different levels, while the Index of well being had only one item. The total score of the Index of Well being and the Index of General Affect was calculated by adding the average scores of its two parts (weight 1.1), with scores ranging from 2.1 to 14.7. In this study, the average subjective well being score was 12.30, and the standard deviation was 3.18.

#### Common method biases

All measurement items were processed by non-rotational exploratory factor analysis, applying the Harman single-factor test method. Based on the results of the analysis, a total of 3 common factors with eigenvalues greater than 1 were extracted, and the first common factor could be used to explain 38.77% of the total variation, which did not reach the standard threshold of 40%. Thus, this study has no unacceptable deviation caused by the same method for data collection (Podsakoff et al., 2003).

## Results

### Descriptive statistics and correlation coefficients of variables

Table 2 shows the results of descriptive statistics and correlation data of the research variables. Bullying victimization not only shows significant positive correlation with aggressive behavior, depression, and anxiety, but also shows significant negative correlation with perceived social support and subjective well being. Aggressive behavior not only shows a significant positive correlation with depression and anxiety, but also shows a significantly negative correlation with perceived social support and subjective well being. Perceived social support shows a significant positive correlation with subjective well being, and a significant negative correlation with depression and anxiety. Moreover, depression is positively correlated with anxiety, and negatively correlated with subjective well being. Last, anxiety has a significant negative correlation with subjective well being.

**Table 2:** Correlation analysis of study variables.

**Variables**	***M***	**SD**	**1**	**2**	**3**	**4**	**5**	**6**
1. Bullying victimization	1.25	0.54	–					
2. Aggressive Behavior	1.04	0.13	0.264**	–				
3. Perceived Social Support	5.05	1.10	−0.361**	−0.198**	–			
4. Depression	3.05	4.56	0.437**	0.253**	−0.414**	–		
5. Anxiety	2.98	3.94	0.415**	0.269**	−0.394**	0.856**	–	
6. Subjective well being	12.30	3.18	−0.320**	−0.211**	0.465**	−0.488**	−0.469**	–

M, Mean; SD. standard deviation.

^**^P <0.01.

### Bullying victimization and mental health: Chain mediating effect test

Chain mediational analysis explored the impact of bullying victimization on mental health through aggressive behavior and perceived social support. Bootstrapping analyses (5,000 re-samples) were conducted for testing the mediational model (Hayes, 2013).

The results showed that the total effect (βs = 0.456, 0.434, and −0.336, *t*s = 29.287, 27.522, and −20.375, All *p* < 0.001) and the direct effect (βs = 0.318, 0.295, and −0.162, *t*s = 19.722, 18.041, and −9.822, All *p* < 0.001) of bullying victimization on depression, anxiety, and subjective well being were all significant. Bullying victimization significantly predicts aggressive behavior (β = 0.264, *t* = 16.507, *p* < 0.001), and aggressive behavior significantly predicts depression, anxiety, and subjective well being (βs = 0.118, 0.142, and –.093, *t*s = 7.992, 9.531, and −6.169, All *p* < 0.001), indicating that aggressive behavior played a mediating role between bullying victimization and depression, anxiety, and subjective well being separately. Similarly, bullying victimization significantly predicts perceived social support (β = −0.332, *t* = 20.800, *p* < 0.001), and perceived social support predicts depression, anxiety, and subjective well being (βs = −0.281, −0.264, and 0.391, *t*s = −18.452, −17.149, and 25.245, All *p* < 0.001), indicating that perceived social support played a mediating role between bullying victimization and depression, anxiety, and subjective well being separately. Meanwhile, aggressive behavior can also predict perceived social support (β = −0.110, *t* = −6.898, *p* < 0.001). Therefore, aggressive behavior and perceived social support had a chain mediating effect between bullying victimization and depression, anxiety, and subjective well being separately among Chinese teenagers (Tables 3–**6**).

**Table 3:** Regression model of the effect of bullying victimization on mental health among Chinese teenagers.

**Variables**	**β**	***t***	***p***	**LLCI**	**ULCI**	***R^2^***	***F***
**Step 1 Outcome variable: Aggressive behavior**						
Predictor bullying victimization	0.264	16.507	<0.001	0.250	0.317	0.070	272.477
**Step 2 Outcome variable: Perceived social support**						
Predictor bullying victimization	−0.332	−20.800	<0.001	−0.356	−0.295	0.141	298.850
Mediator aggressive behavior	−0.110	−6.898	<0.001	−0.129	−0.072		
**Step 3 Outcome variable: Depression**						
Predictor bullying victimization	0.305	19.722	<0.001	0.286	0.350	0.279	468.533
Mediator 1 aggressive behavior	0.118	7.992	<0.001	0.086	0.142		
Mediator 2 Perceived social support	−0.281	−18.452	<0.001	−0.330	−0.267		
**Step 4 Outcome variable: Anxiety**						
Predictor bullying victimization	0.283	18.041	<0.001	0.263	0.327	0.260	424.617
Mediator 1 aggressive behavior	0.142	9.531	<0.001	0.110	0.167		
Mediator 2 Perceived social support	−0.264	−17.149	<0.001	−0.313	−0.249		
**Step 5 Outcome variable: Subjective** well being						
Predictor bullying victimization	–.155	−9.822	<0.001	–.195	–.130	0.251	405.768
Mediator 1 aggressive behavior	–.093	−6.169	<0.001	–.119	–.062		
Mediator 2 Perceived social support	.391	25.245	<0.001	.386	.451		

**Figure 2:**
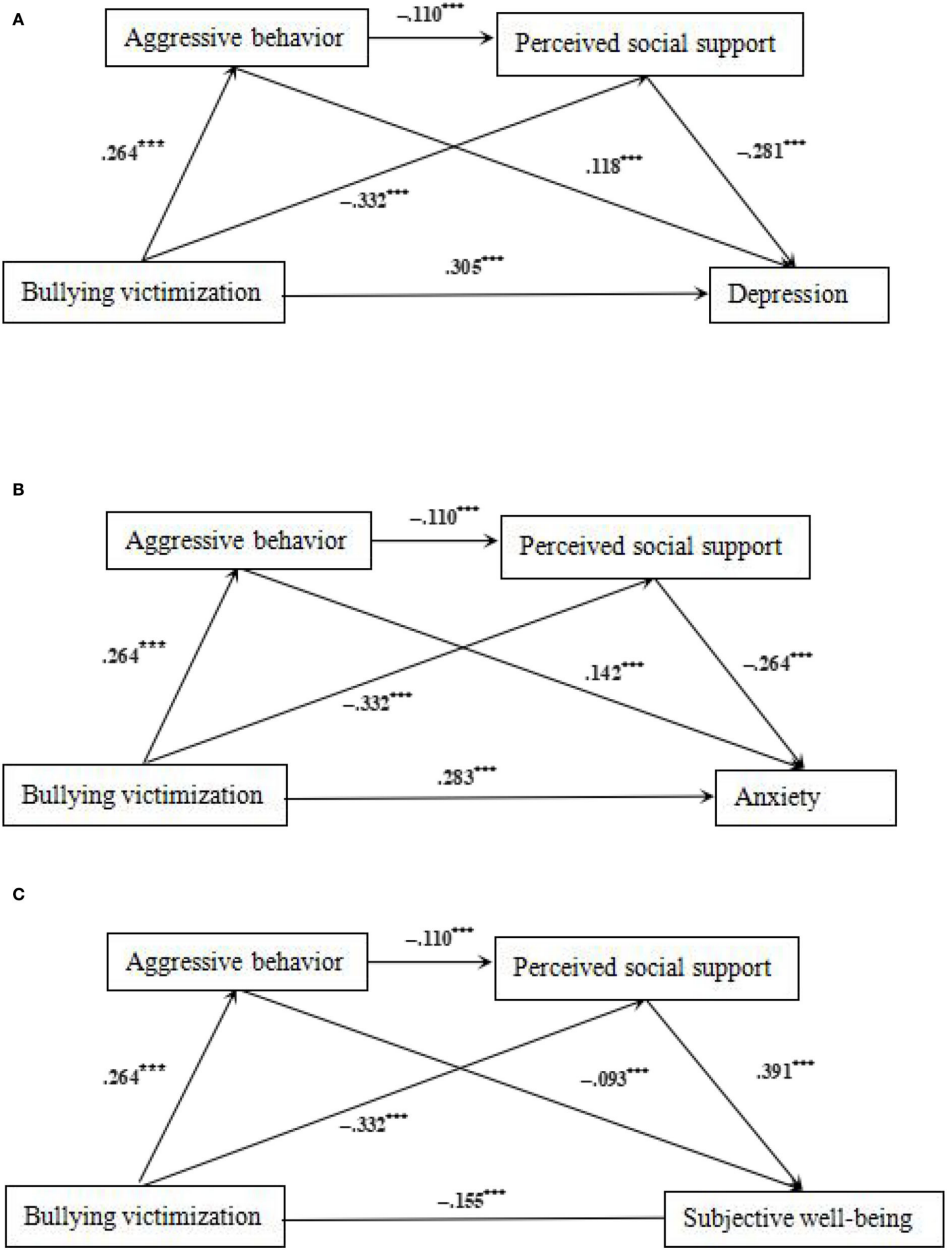
**(A)** The mediating effect path map of bullying victimization and depression. *p < 0.05, **p < 0.01, and ***p < 0.001. **(B)** The mediating effect path map of bullying victimization and anxiety. *p < 0.05, **p < 0.01, and ***p < 0.001. **(C)** The mediating effect path map of bullying victimization and subjective well being. *p < 0.05, **p < 0.01, and ***p < 0.001.

Results of the mediating effect analysis of bullying victimization and depression, anxiety, and subjective well being in Tables 4–6 showed that Bootstrap's 95% CI of total indirect effect did not contain 0 [All Bootstrap 95% CI: 0.114, 0.166; 0.115, 0.165; −0.202, −0.149], accounting for 30.26, 32.03, and 51.79% of the total effect. Notably, three indirect effect pathways influenced the relation of bullying victimization and depression, anxiety, and subjective well being. First, the mediating effect values of Path1 (Bullying victimization → Aggressive behavior → Depression, Anxiety, and Subjective well being) were 0.032, 0.039, and −0.026 separately, accounting for 7.02, 8.99, and 7.74% of the total effect. Second, the mediating effect values of Path2 (Bullying victimization → Perceived social support → Depression, Anxiety, and Subjective well being) were 0.097, 0.092, and −0.136 separately, accounting for 21.27, 21.20, and 40.48% of the total effect. Third, the mediating effect values of Path3 (Bullying victimization → Aggressive behavior → Perceived social support → Depression, Anxiety, and Subjective well being) was 0.009, 0.008, and −0.012 separately, accounting for 1.97, 1.84, and 3.57% of the total indirect effect. Note that the chain mediating model is shown in Figures 2A–C.

**Table 4:** Mediating effect analysis of bullying victimization and depression.

	**Effect**	**Boot**	**Bootstrap**	**Effect**
		**SE**	**95% CI**	**ratio**
Total effect	0.456	0.016	[0.425, 0.486]	100%
Direct effect	0.318	0.016	[0.286, 0.350]	69.74%
Total indirect effect	0.138	0.013	[0.114, 0.166]	30.26%
Path 1: Bullying victimization → Aggressive behavior → epression	0.032	0.008	[0.019, 0.050]	7.02%
Path 2: Bullying victimization → Perceived social support → Depression	0.097	0.011	[0.077, 0.118]	21.27%
Path 3: Bullying victimization → Aggressive behavior → Perceived social support → Depression	0.009	0.002	[0.005, 0.014]	1.97%

**Table 5:** Mediating effect analysis of bullying victimization and anxiety.

	**Effect**	**Boot**	**Bootstrap**	**Effect**
		**SE**	**95% CI**	**ratio**
Total effect	0.434	0.016	[0.403, 0.465]	100%
Direct effect	0.295	0.016	[0.263, 0.327]	67.97%
Total indirect effect	0.139	0.013	[0.115, 0.165]	32.03%
Path 1: Bullying victimization → Aggressive behavior → Anxiety	0.039	0.008	[0.025, 0.056]	8.99%
Path 2: Bullying victimization → Perceived social support → Anxiety	0.092	0.010	[0.073, 0.112]	21.20%
Path 3: Bullying victimization → Aggressive behavior → Perceived social support → Anxiety	0.008	0.002	[0.004, 0.013]	1.84%

**Table 6:** Mediating effect analysis of bullying victimization and subjective well being.

	**Effect**	**Boot**	**Bootstrap**	**Effect**
		**SE**	**95% CI**	**ratio**
Total effect	−0.336	0.017	[−0.368, −0.304]	100%
Direct effect	−0.162	0.017	[−0.195, −0.130]	48.21%
Total indirect effect	−0.174	0.014	[−0.202, −0.149]	51.79%
Path1: Bullying victimization → Aggressive behavior → Subjective well being	−0.026	0.005	[−0.036, −0.017]	7.74%
Path2: Bullying victimization → Perceived social support → Subjective well being	−0.136	0.012	[−0.161, −0.113]	40.48%
Path3: Bullying victimization → Aggressive behavior → Perceived social support → Subjective well being	−0.012	0.003	[−0.019, −0.007]	3.57%

## Discussion

The initial objective of the research was to identify the impact of bullying victimization on mental health, and the serial mediating roles of aggressive behavior and perceived social support among adolescents. As indicated by the results of this study, the experience of being bullied significantly increased the level of mental health issues, such as higher levels of anxiety and depression, and lower levels of subjective well being. The relationship results are consistent with previous studies and verified Hypothesis 1 in the study (Juvonen and Graham, 2014).

The present study also discovered that aggressive behavior as a maladaptive reaction had significant mediating effects on the relationship between the experience of being bullied and mental health, with its mediating role accounting for 7.02, 8.99, and 7.74% of the total effect for anxiety, depression, and subjective well being. The Hypothesis 2 is confirmed. In addition, these results verified the negative outcome of aggressive behavior for bullying victims, which demonstrated that bullying victims' crude responses would aggravate the mental health issue. Violence begets violence provides no constructive solutions.

Meanwhile, the current study also found significant mediating effects of perceived social support in the relationship between the experience of being bullied and mental health, with its mediating effect accounting for 21.27, 21.20, and 40.48% of the total effect for anxiety, depression, and subjective well being. The present results are congruent with the latest research in the area of bullying victims and prove Hypothesis 3 (Lin et al., 2020). Perceived social support from parents, friends, and other relatives is a vital protective factor to disengage bullying victims from mental health issues.

Previous studies do not adequately consider the multiple coping strategies of bullying victims simultaneously and examine the underlying relationship among these mechanisms. The current study explored the impact of bullying victims' aggressive behavior to perceived social support, and whether aggressive behavior and perceived social support serially mediated the relationship between bullying victimization and mental health. The aggressive behavior of bullying victims may produce feelings of isolation in interpersonal relationships and is harmful for victims' mental health status. The results support Hypothesis 4 that the higher level of bullying victimization would raise the possibility of mental health issues through maladaptive aggressive behavior and lower the level of perceived social support. It has also been confirmed that there is a close correlation between aggressive reaction and perceived social support (Yang, 2020). The current study's outcome revealed the basic psychological processes of an individual being bullied and helped us in understanding how their variety of responses led to disastrous outcomes. Teenager bullying victims may adopt simple and rough maladaptive behavior, which aggravate the individual's mental health problems. This process will also weaken the individual's perception of positive resources. This mechanism is a kind of interlocking “Tragic Chain”.

The research revealed the process of how bullying victim's maladaptive coping strategies generates mental health issues through aggressive behavior and perceived social support in a large sample of Chinese adolescents. It is a risk factor for the mental health development of teenagers with the experience of being bullied. That is, the impulsive aggressive behavior of bullying victims would reduce their perceived social support and put their mental health status in danger. It implied that a violent response to violence produces chains of tragedy in bullying situations. Recent research in the related area also discovered the similar phenomenon that forgiving rather than revenge can regain the feeling of humanity after the victimization experience (Schumann and Walton, 2022).

Furthermore, the results of the present study support the implementation of bully prevention programs and actions, including: enhancing individual strategies effectively counteract bullying, and increasing empathy toward victims; attaching importance to the social support from peers, school staff, parents, and other stakeholders, guide them to improve assistance afforded to victims, and other relative interventions (Salmivalli et al., 2011; Roca-Campos et al., 2021). The mediating chain effect of the study sheds light on the underlying processes that the victim's maladaptive behavior would reduce the perceived social support, and then deteriorate mental health. The increasing understanding of these processes supports detailed application to reduce the insensitivity of social resources and mental health problems caused by the experience of being bullied and, through modifying the maladaptive aggressive behavior, alleviates the bullying's negative influence to a certain extent, then breaks the chains of tragedy.

## Limitations and future orientation

The current research has some deficiencies and limitations, which may be addressed in the future. First, the present study was a cross-sectional design study, it cannot clarify the causal relationship between variables. Therefore, further research should be conducted to better clarify the relationship between variables through experimental design and longitudinal study. Second, the current research results are in the context of Chinese cultural background. In a collective culture, individuals' survival and development needs depend more on interpersonal relations (Yum, 1988). Future studies could explore whether there is a cultural difference in the mechanism of maladaptive reactions of bullying victims.

## Conclusion

Bullying and victimization is a growing area of research in psychology. The present study provides further scientific evidence for intervention after the experience of being bullied at the behavioral and cognitive levels. The Chains of Tragedy of bullying victims who conduct maladaptive behavior would lead to a change in their perceived social support and mental health problems, reminding us to draw attention to the sequential effect of multiple variables at work in bully prevention.

## Data availability statement

The raw data supporting the conclusions of this article will be made available by the authors, without undue reservation.

## Ethics statement

The studies involving human participants were reviewed and approved by Ethics Committee of Hubei University of Education. Written informed consent to participate in this study was provided by the participants' legal guardian/next of kin.

## Author contributions

YG: conceptualization, collected the data, writing—review and editing, data curation, and worked on the final version of the manuscript. XT: writing—review and editing, data curation, and worked on the final version of the manuscript. QZ: conceptualization, collected the data, writing—formal analysis, data curation, and worked on the final version of the manuscript. All authors contributed to the article and approved the submitted version.

## Funding

This work was supported by Ministry of Education of the People's Republic of China Humanities and Social Sciences Youth Foundation under Grant (No. 19YJCZH044).

## Conflict of interest

The authors declare that the research was conducted in the absence of any commercial or financial relationships that could be construed as a potential conflict of interest.

## Publisher's note

All claims expressed in this article are solely those of the authors and do not necessarily represent those of their affiliated organizations, or those of the publisher, the editors and the reviewers. Any product that may be evaluated in this article, or claim that may be made by its manufacturer, is not guaranteed or endorsed by the publisher.
